# A new species of *Anomognathus* and new Canadian and provincial records of aleocharine rove beetles from Alberta, Canada (Coleoptera, Staphylinidae, Aleocharinae)

**DOI:** 10.3897/zookeys.581.8014

**Published:** 2016-04-14

**Authors:** Jan Klimaszewski, David W. Langor, H.E. James Hammond, Caroline Bourdon

**Affiliations:** 1 Natural Resources Canada, Canadian Forest Service, Laurentian Forestry Centre, 1055 du P.E.P.S., P.O. Box 10380, Stn. Sainte-Foy, Québec, Quebec, Canada G1V 4C7; 2 Natural Resources Canada, Canadian Forest Service, Northern Forestry Centre, 5320-122 Street, Edmonton, Alberta, Canada T6H 3S5

**Keywords:** Coleoptera, rove beetles, Staphylinidae, Aleocharinae, new provincial records, new species, Canada, Alberta

## Abstract

A new species, *Anomognathus athabascensis* Klimaszewski, Hammond & Langor, **sp. n.**, and nine new provincial records including one new country record of aleocharine beetles are presented for the province of Alberta. Diagnostics, images of habitus and genital structures, distribution, natural history information and new locality data are provided for the newly recorded species. A checklist for all recorded aleocharines from Alberta is updated.

## Introduction

A survey of beetles from several localities, mainly in the Athabasca region of Alberta, was conducted in 1997 by J. Hammond and D. Langor of the Canadian Forest Service, Northern Forestry Centre. As a result, 33 species of rove beetles were identified. Of these, 29 belong to aleocharines and 5 to other families of Staphylinidae (*Anotylus* sp., *Carpelimus* sp., *Heterothops minor* Smetana, *Phloeonoma laesicollis* Mäklin and *Phloeostiba lapponica* Zetterstedt). Among the aleocharines, we discovered one species new to science, *Anomognathus athabascensis*, the second known species of this genus from North America, as well as one new country and eight new provincial distribution records for species known in other parts of Canada (Table 1).

**Table 1.** Species of Aleocharinae recorded from Alberta, and their provincial and territorial distribution within Canada. Provinces and territories in bold denote new records given in the present publication. Species marked with (†) indicate adventive species and species marked with (*) are Holarctic.

ALEOCHARINI	
*Aleochara bilineata* Gyllenhal†	AB, BC, MB, NB, NF, NS, ON, PE, QC, SK
*Aleochara bimaculata* Gravenhorst	AB, BC, LB, MB, NB, NF, NS, ON, QC, SK, NT
*Aleochara castaneipennis* Mannerheim	AB, BC, LB, NB, NF, NS, NT, ON, QC, YT; USA: AK
*Aleochara fumata* Mannerheim	AB, BC, LB, NB, NF, NS, NT, ON, QC, YT; USA: AK
*Aleochara lacertina* Sharp	AB, BC, MB, NB, NF, NS, ON, QC, SK
*Aleochara lanuginosa* Gravenhorst†	AB, BC, MB, NF, NB, NS, ON, QC, SK
*Aleochara sekanai* Klimaszewski	AB, LB, MB, NB, NT, ON, SK, YT; USA: AK
*Aleochara speculicollis* Bernhauer	AB, ON, QC
*Aleochara suffusa* (Casey)	AB, BC, MB, QC; USA: AK
*Aleochara tahoensis* Casey	AB, BC, MB, NB, NS, NT, ON, SK, YT
*Aleochara verna* Say	AB, BC, LB, MB, NB, NF, NS, ON, PE, QC, SK, YT; USA: AK
*Aleochara villosa* Mannerheim†	AB, BC, NB, QC
*Tinotus morion* (Gravenhorst)†	AB, BC, NB, NF, NS, ON, QC, SK; USA: CT, NV
**ATHETINI**	
***Atheta borealis* Klimaszewski & Langor**	**AB**, NF
*Atheta dadopora* C.G. Thomson*	AB, BC, LB, NB, NF, NS, ON, PE, SK, YT; USA: AK, NY, PA, RI
*Atheta districta* Casey	AB, BC, LB, NB, NF, NS, ON, QC
*Atheta fanatica* Casey	AB, BC, LB, NB, NS, QC, SK, YT; USA: AK, NV
*Atheta graminicola* (Gravenhorst)*	AB, BC, LB, MB, NB, NF, NT, ON, QC, SK, YT; USA: AK, OR
***Atheta hampshirensis* Bernhauer**	**AB**, BC, NB, NF, NS, ON, QC; USA: AK, CA, NC, NH, NY, OR, PA, RI, WA
*Atheta klagesi* Bernhauer	AB, NB; USA: ME, PA [all other previously published records of this species need to be revised]
*Atheta modesta* (Melsheimer)	AB, NB, NS, ON, QC; USA: CT, DC, MI, NY, PA, RI, VA, VT
*Atheta platonoffi* Brundin*	AB, BC, LB, NB, NF, NS, ON, SK, YT; USA: AK
***Atheta pseudoklagesi* Klimaszewski &Webster**	**AB**, NB [all published records of *Atheta klagesi* need to be revised because they may contain mixed series with *Atheta pseudoklagesi*]
*Atheta pseudosubtilis* Klimaszewski & Langor	AB, LB, NB, NF, QC
*Atheta remulsa* Casey	AB, BC, LB, NB, NF, NS, ON, QC, YT
*Atheta ventricosa* Bernhauer	AB, BC, LB, NB, NF, NS, ON, QC, SK, YT; USA: AK, DC, NC, NJ, NY, PA, VT
*Boreophilia davidgei* Klimaszewski & Godin	AB, YT
*Boreophilia islandica* (Kraatz)*	AB, NF, NT, NU, YT; USA: AK
*Boreostiba parvipennis* (Bernhauer)	AB, LB, NF, NT, QC, YT; USA: AK, NH
*Dalotia coriaria* (Kraatz)†	AB, BC, NB, NS, ON; USA: LA, NY
*Dinaraea angustula* (Gyllenhal)†	AB, LB, NB, NF, NS, ON, PE, QC, YT; USA: CA, NY
*Dinaraea pacei* Klimaszewski & Langor	AB, BC, LB, NB, QC, YT; USA: AK
*Dinaraea worki* Klimaszewski & Jacobs	AB, QC
*Earota dentata* (Bernhauer)	AB, BC, MB, NB, NF, NS, ON, QC, YT; USA: AK
*Liogluta aloconoides* Lohse	AB, LB, NF, NS, YT
*Lypoglossa franclemonti* Hoebeke	AB, MB, NB, NF, NS, NT, ON, QC, SK, YT; USA: NY, VT
*Mocyta breviuscula* (Mäklin)	AB, BC, LB, NB, NF, NS, ON, QC, YT; USA: AK, OR
*Mocyta fungi* (Gravenhorst)†	AB, BC, LB, NB, NF, NS, NU, ON, PE, QC, SK, YT: USA: AK
*Paragoniusa myrmicae* Maruyama & Klimaszewski	AB, BC, LB
*Philhygra botanicarum* (Muona)*	BC, LB, NB, NF, NS, ON, SK, YT
*Philhygra satanas* (Bernhauer)	AB; USA: CA
*Philhygra sinuipennis* Klimaszewski & Langor	NB, LB, NF, SK, YT
***Philhygra subpolaris* (Fenyes)**	**AB**; USA: AZ
*Schistoglossa campbelli* Klimaszewski	AB, BC
*Schistoglossa hampshirensis* Klimaszewski	AB, NB, QC; USA: NH
*Seeversiella globicollis* (Bernhauer)	AB, BC, NB, NF, NS, ON, QC, SK; USA: AZ, CO, ID, MN, MT, NH, SD, WI; Mexico; Guatemala
*Strophogastra pencillata* Fenyes	AB, MB, NB, NS, ON, QC
*Trichiusa pilosa* Casey	AB, BC, NS, ON; USA: ID, IN, KS, OH, RI
**AUTALIINI**	
*Autalia rivularis* (Gravenhorst)†	AB, BC, LB, NB, NF, NS, ON, QC
**FALAGRINI**	
*Falagria caesa* Erichson†	AB, NB, ON, QC
*Falagria dissecta* Erichson	AB, BC, MB, NB, NS, ON, QC; across USA
**GYMNUSINI**	
*Gymnusa atra* Casey*	AB, BC, LB, MB, NB, NF, NS, NT, NU, ON, QC, YT; USA: AK
*Gymnusa pseudovariegata* Klimaszewski	AB, BC, LB, MB, NB, NF, NS, NT, ON, QC, YT; USA: AK
**HOMALOTINI**	
***Agaricomorpha vincenti* Klimaszewski & Webster**	**AB**, NB
***Anomognathus athabascensis* Klimaszewski, Hammond & Langor , sp. n.**	**AB**
*Gyrophaena keeni* Casey	AB, BC, LB, NB, NF, ON, QC, YT; USA: FL, MA, MT, NH, NY, TN, WA, WI
*Gyrophaena modesta* Casey	AB, NB, NF, NS, ON; USA: IL, IN, MI, MN, NH
*Gyrophaena nana* (Paykull)*	AB, BC, MB, NB, NF, NS, ON; USA: MA, ME, MT, WI, WY
***Gyrophaena sculptipennis* Casey**	**AB**, NB, NS, ON, QC; USA : MA, NH, NY, WI
*Gyrophaena uteana* Casey	AB, BC, NB, ON, QC, SK; USA: CA, CO, UT
*Gyrophaena wisconsinica* Seevers	AB, NB, QC; USA: WI
*Homalota plana* (Gyllenhal)†	AB, NB, NF, NS; USA: AK; Palaearctic: Europe, Asia
*Leptusa gatineauensis* Klimaszewski & Pelletier	AB, BC, NB, NF, NS, ON, QC
*Neotobia albertae* Ashe	AB, MB, NB, ON, QC
*Phymatura blanchardi* (Casey)	AB, NB, ON
*Silusa californica* Bernhauer	AB, BC, LB, NB, NF, NS, ON, PE, QC, YT; USA: AK, CA, MN
*Silusa densa* Fenyes	AB, LB, NB, NF; USA: CA
*Silusa langori* Klimaszewski	AB, NB
**LOMECHUSINI**	
*Pella criddlei* (Casey)	AB, MB, QC
*Pella gesneri* Klimaszewski	AB, MB, NB, ON
*Xenodusa reflexa* (Walker)	AB, BC, MB, NB, NS, QC, ON, SK
**MYLLAENINI**	
*Myllaena arcana* Casey	AB, LB, NB, NF, NS, ON, QC, SK; USA: AL, FL, IA, IL, MA, NH, NJ; Mexico
*Myllaena insomnis* Casey	AB, BC, LB, MB, NB, NF, NS, NT, ON, QC, SK, YT; USA: AK, ID, MA, MN, WI
**OXYPODINI**	
*Devia prospera* (Erichson)*	AB, BC, LB, MB, NB, NT, ON, SK, YT; USA: AK, CO, MI, MN, NM, OR, SD, UT, WA, WY
*Gnathusa eva* Fenyes	AB, BC, YT; USA: CA
*Gnathusa tenuicornis* Fenyes	AB, BC, NB, YT; USA: CA, OR
*Gnypeta caerula* (C.R. Sahlberg)*	AB, BC, LB, MB, NB, NF, NS, NT, ON, PE, QC, SK, YT; USA: AK
*Gnypeta canadensis* Klimaszewski	AB, ON
*Gnypeta carbonaria* (Mannerheim)	AB, MB, NB, NF, NT, ON, QC, SK; USA: AK
*Gnypeta helenae* Casey	AB, BC, ON
***Hylota cryptica* Klimaszewski & Webster**	**AB**, NB
*Oxypoda canadensis* Klimaszewski	AB, LB, MB, NF, NT, ON, QC, YT; USA: AK
*Oxypoda convergens* Casey	AB, LB, NB, NF, NS, ON, QC; USA: IA, MO, NY
*Oxypoda frigida* Bernhauer	AB, BC, LB, NF, NB, NS, NT, ON, QC, YT; USA: AK
*Oxypoda grandipennis* (Casey)	AB, BC, LB, NB, NF, NS, ON, QC, SK, YT; USA: AK, NH
*Oxypoda hiemalis* Casey	AB, LB, NB, NF, NS, NT, ON, QC; USA: AK
*Oxypoda lacustris* Casey	AB, BC, LB, MB, NB, NF, NS, NT, ON, QC, SK, YT; USA: AK
*Oxypoda lucidula* Casey	AB, LB, MB, NB, NF, NT, ON, QC, YT; USA: AK, IA, MO, NH, NY
*Oxypoda operta* Sjöberg†	AB, LB, NS, ON, QC, YT; USA: NH
*Oxypoda orbicollis* Casey	AB, LB, NB, NS, ON, QC, SK, YT; USA: WI
*Oxypoda pseudolacustris* Klimaszewski	AB, NB, NF, NS, ON, QC, SK
*Tachyusa americanoides* Casey	AB, BC, MB, NB, NF, NS, NT; USA : IL, MA, NH, NY
**PLACUSINI**	
*Placusa incompleta* Sjöberg†	AB, BC, NB, NF, NS, ON, QC; USA: WA
*Placusa pseudosuecica* Klimaszewski	AB, BC, ON, QC
*Placusa tachyporoides* (Waltl)†	AB, BC, NB, NS, ON, QC
*Placusa tacomae* Casey	AB, BC, NB, NF, NS, NT, ON, QC, YT; USA: AZ, MA, WA, WI
***Placusa vaga* Casey**	**AB**, BC, NB, NS, NT, ON, QC, YT; USA: CA
**96 species, 9** new records including one new country record and one new species.	7 adventive and 4 Holarctic species

These findings are reported together with an updated checklist of all species from the province (Table 1). The previous lists were published by Bousquet et al. (2013), Gouix and Klimaszewski (2007), and Klimaszewski et al. (2015).

## Materials and methods

All specimens in this study were dissected to examine the genital structures. Extracted genital structures were dehydrated in absolute alcohol, mounted in Canada balsam on celluloid micro-slides, and pinned with the specimens from where they originated. Images of the entire body and the genital structures were taken using an image processing system (Nikon SMZ 1500 stereoscopic microscope; Nikon Digital Camera DXM 1200F, and Adobe Photoshop software).

Morphological terminology mainly follows that used by Seevers (1978) and Klimaszewski et al. (2011). The ventral side of the median lobe of the aedeagus is considered to be the side of the bulbus containing the foramen mediale, the entrance of the ductus ejaculatorius, and the adjacent ventral side of the tubus of the median lobe with the internal sac and its structures (this part is referred to as the parameral side in some recent publications); the opposite side is referred to as the dorsal part. In the species descriptions, microsculpture refers to the surface of the upper forebody (head, pronotum and elytra).

### Depository/institutional abbreviations

LFC
 Natural Resources Canada, Canadian Forest Service, Laurentian Forestry Centre, R. Martineau Insectarium, Québec, Canada 


NoFC
 Natural Resources Canada, Canadian Forest Service, Northern Forestry Centre, Arthropod Museum, Edmonton, Alberta, Canada 


### Abbreviations of Canadian provinces and territories

AB – Alberta


BC – British Columbia


LB – Labrador


MB – Manitoba


NB – New Brunswick


NF – Newfoundland


NS – Nova Scotia


NT – Northwest Territories


NU – Nunavut


ON – Ontario


PE – Prince Edward Island


QC – Quebec


SK – Saskatchewan


YT – Yukon Territory


### Discussion

A new study of aleocharine rove beetles from Alberta revealed one subcortical species new to science, and eight other species representing new provincial records, including one new to Canada. A checklist of aleocharine species from Alberta, including present data, indicates 96 species classified in nine tribes. Of these, 78 are considered to be native species, six Holarctic and 12 adventive (Table 1). The total number of 96 species is very low in comparison with the eastern provinces and reflects poor knowledge of this group in Alberta. The true number of aleocharines in Alberta remains unknown but it is anticipated to be comparable to or surpass that of Newfoundland and Labrador, currently estimated at 189 species (Klimaszewski et al. 2011, and unpublished data). New taxonomic inventories are badly needed to provide baseline taxonomic data by which to assess change due to anthropogenic and natural disturbances and climate change.

## Taxonomic review

### ATHETINI Casey

#### Atheta (s. str.) borealis

Taxon classificationAnimaliaColeopteraStaphylinidae

Klimaszewski & Langor

Figs 1–4

Atheta (s. str.) borealis Klimaszewski & Langor, in Klimaszewski et al. 2011: 116.

##### Diagnosis.

This species may be distinguished from other Nearctic *Atheta* (s. str.) by its uniformly black and glossy body, sparse pubescence of forebody, antennal articles elongate, and the shape of its genital structures (Figs 2–4). For a detailed description, see Klimaszewski et al. (2011).

**Figures 1–4.**
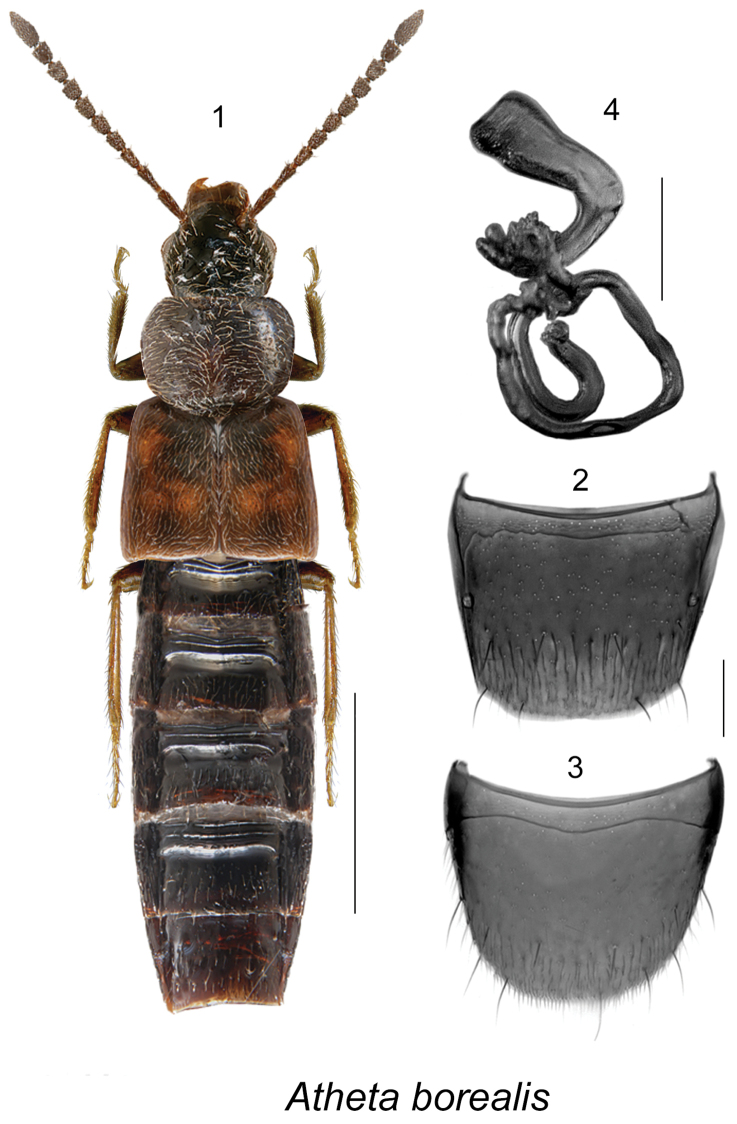
Atheta (s. str.) borealis Klimaszewski & Langor (female): **1** habitus in dorsal view **2** tergite VIII **3** sternite VIII **4** spermatheca. Scale bar of habitus = 1 mm; remaining scale bars = 0.2 mm.

##### Distribution.

**Table:** 

Origin	Nearctic
Distribution	Canada: NF, **AB**
New records	New provincial record: **Canada, Alberta**: Slave Lake, 4 km SW Mitsue Lake, 55.2080°N, 114.6789°W, Hammond window-trap, H-68-3-6 (SL), 1997.08.11 (NoFC) 1 female
Reference	Klimaszewski et al. 2011

##### Natural history.

Very little is known about the life history of this species. Adults in Newfoundland were captured in pitfall traps on a coastal limestone barren and in riparian forest (Klimaszewski et al. 2011). The Alberta specimen was captured in a window-trap attached to aspen snag in boread aspen forest harvested 29 years previously. Adults were collected in August in Alberta and Newfoundland.

##### Comments.

This species is likely continuously distributed in northern boreal forest of Canada.

#### Atheta (Dimetrota) hampshirensis

Taxon classificationAnimaliaColeopteraStaphylinidae

Bernhauer

Figs 5–12

Atheta (Dimetrota) hampshirensis Bernhauer 1909: 525, Gusarov 2003: 43, Klimaszewski et al. 2011: 139.

##### Diagnosis.

**Figures 5–12.**
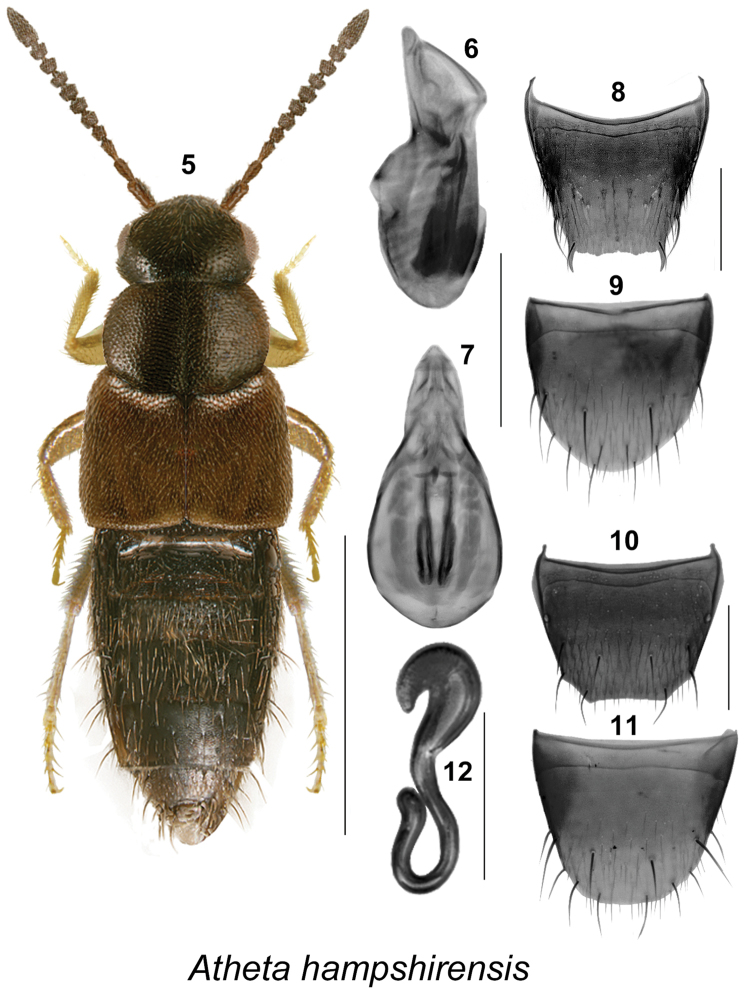
Atheta (Dimetrota) hampshirensis Bernhauer: **5** habitus in dorsal view **6** median lobe of aedeagus in lateral view **7** median lobe of aedeagus in dorsal view **8** male tergite VIII **9** male sternite VIII **10** female tergite VIII **11** female sternite VIII **12** spermatheca. Scale bar of habitus = 1 mm; remaining scale bars = 0.2 mm.

This species may be distinguished from other Nearctic Atheta (Dimetrota) by its small size (length 2.2–2.6 mm), uniformly black body, dense and asperate punctation of forebody, antennal articles slightly to strongly transverse (Fig. 5), and the shape of its genital structures (Figs 6–12). For a detailed description, see Klimaszewski et al. (2011).

This species may be confused with *Atheta dadopora* Thomson and *Strophogastra pencillata* Fenyes. *Strophogastra pencillata* differs from *Atheta hampshirensis* by having numerous strong ventral setae near the apical part of the abdomen and *Atheta dadopora* is more elongate and has different body proportions. All three species differ in the shape of male tergite VIII, median lobe of aedeagus and spermatheca.

##### Distribution.

**Table:** 

Origin	Nearctic
Distribution	Canada: NF, NS, NB, QC, ON, **AB**, BC. USA: AK, CA, NC, NH, NY, OR, PA, RI, WA
New records	New provincial record: **Canada, Alberta**: Smith, 10 km N Lawrence Lake, 55.0432°N, 113.6650°W, Hammond window-trap, H-95-3-1 (LL), 1997.07.16 (NoFC) 1 female
References	Bernhauer 1909, Lohse and Smetana 1985, Klimaszewski and Winchester 2002, Gusarov 2003, Klimaszewski et al. 2005, Webster et al. 2009, Majka and Klimaszewski 2008, 2010, Klimaszewski et al. 2011

##### Natural history.

In Newfoundland, adults were collected from June to August using carrion-baited pitfall traps and flight intercept traps in mixedwood and coniferous forest types and on coastal barrens (Klimaszewski et al. 2011). In British Columbia, adults were taken from Sitka spruce forest, June through September, with peak abundance in August/September (Klimaszewski and Winchester 2002). In New Brunswick, adults were found in red spruce forest from July to September (Klimaszewski et al. 2005), and in Nova Scotia in coniferous and deciduous forests, open habitats, on mushrooms, in compost and on carrion (Majka and Klimaszewski 2008).

The Alberta female was captured in July in a window-trap attached to the trunk of an aspen snag in a two-year-old harvested boreal aspen stand.

##### Comments.

This species is broadly distributed in Canada and the USA.

#### Atheta (Pseudota) pseudoklagesi

Taxon classificationAnimaliaColeopteraStaphylinidae

Klimaszewski & Webster

Figs 13–20

Atheta (Pseudota) pseudoklagesi Klimaszewski & Webster (in Webster et al. 2016: 132)

##### Diagnosis.

**Figures 13–20.**
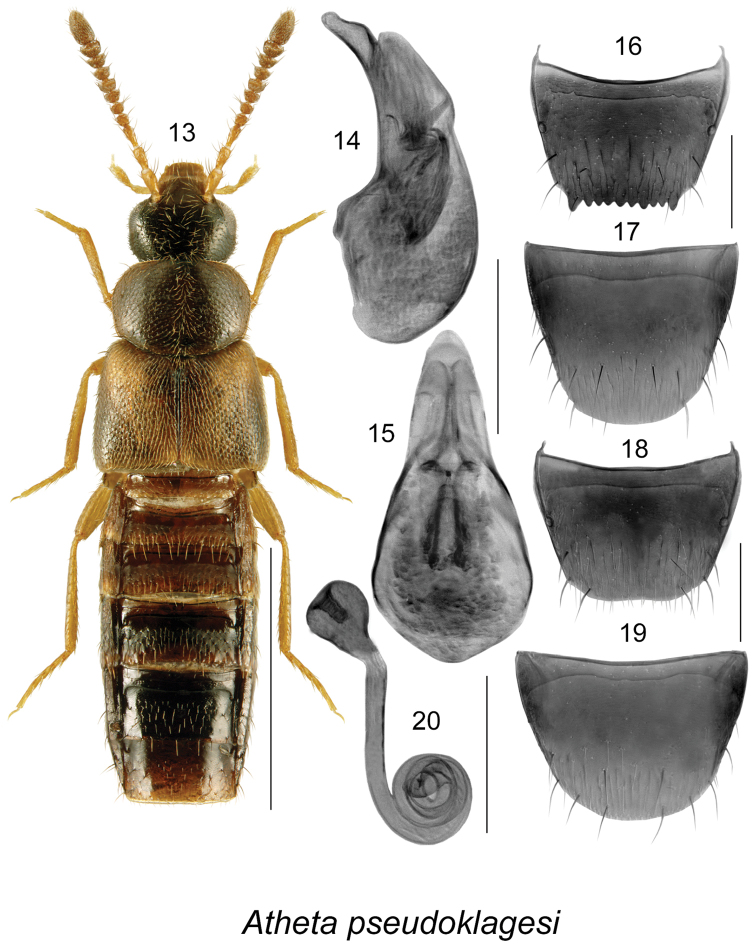
Atheta (Pseudota) pseudoklagesi Klimaszewski & Webster: **13** habitus in dorsal view **14** median lobe of aedeagus in lateral view **15** median lobe of aedeagus in dorsal view **16** male tergite VIII **17** male sternite VIII **18** female tergite VIII **19** female sternite VIII **20** spermatheca. Scale bar of habitus = 1 mm; remaining scale bars = 0.2 mm.

This is a sibling species of *Atheta klagesi* Bernhauer and was frequenly confused with the latter in collections. It may be distinguished from *Atheta klagesi* by its slightly larger size, less glossy body, less intense yellowish colouration of spots on elytra, less intense yellowish colouration of legs, bases of antennae and maxillary palps and overall less contrasting body colour (Fig. 13); median lobe of aedeagus has longer tubus and slightly different shape of apex in lateral view (Fig. 14); spermatheca is very similarly shaped in the two species (Fig. 20), and females may be difficult to identify without accompanying males.

##### Distribution.

**Table:** 

Origin	Nearctic
Distribution	Canada: NB, **AB.** Currently known only from New Brunswick and Alberta, but because of confusion with *Atheta klagesi*. This species will undoubtedly prove to be more widespread.
New records	New provincial record: **Canada, Alberta**: Ft. McMurray, 15 km N Mariana Lake, 56.1848°N, 111.9513°W, Hammond window-trap, F-95-3-1 (FM), 1997.07.09 (NoFC) 1 female; Ft. McMurray, 15 km N Mariana Lake, 56.1848°N, 111.9513W, Hammond window-trap, F-95-3-3 (FM), 1996.08.01 (NoFC) 1 male; same data except – F-95-3-1 (FM), 1997.07.24 (NoFC) 1 male.
Reference	Webster et al. (2016)

##### Natural history.

In New Brunswick, adults of this species were found in mature mixed forest, old-growth and old white spruce and balsam fir forests, a mature red spruce forest, and in a wet alder swamp (Webster et al. 2016). Specimens were collected from coral fungi on *Populus* log, fleshy polypore fungi at base of a dead standing *Populus*, in decaying gilled mushrooms, in gilled mushrooms, and under bark of red spruce (Webster et al. 2016). Adults were collected from May to September. The Alberta specimens were captured in July in a window-trap.

##### Comments.

This species is very likely broadly distributed in Canada and the northern USA, but the existing records for *Atheta klagesi* (except for type series) need to be revised because they may contain mixed series of *Atheta klagesi* and *Atheta pseudoklagesi*.

#### Philhygra subpolaris

Taxon classificationAnimaliaColeopteraStaphylinidae

(Fenyes)

Figs 21–26

Brundinia subpolaris Fenyes 1909: 423.

##### Diagnosis.

**Figures 21–26.**
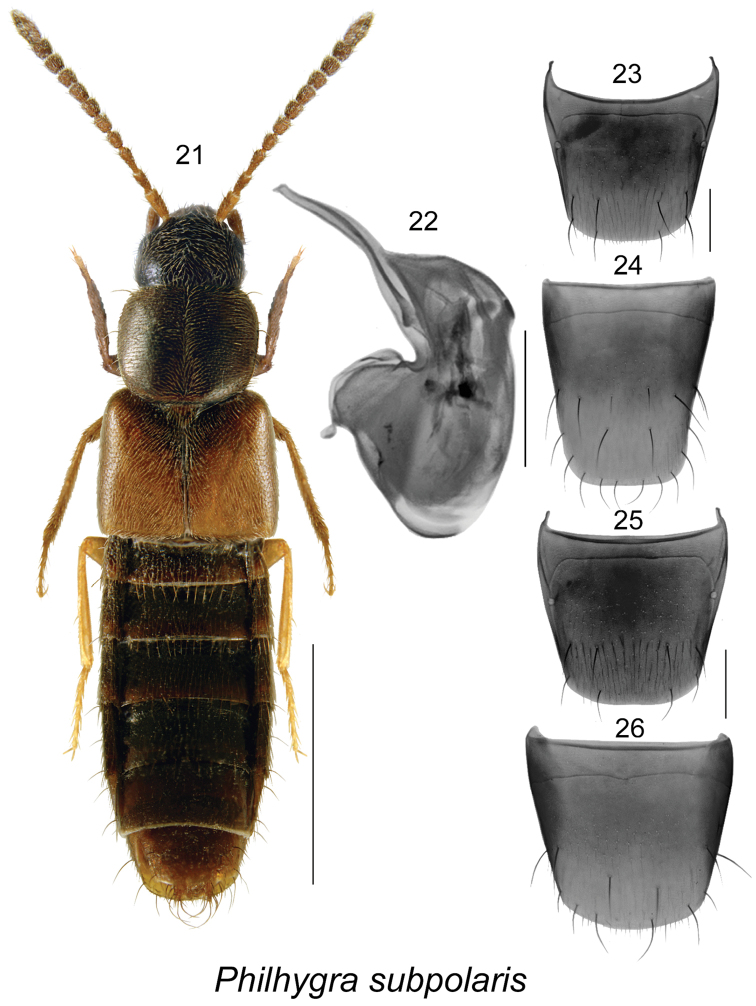
*Philhygra subpolaris* (Fenyes): **21** habitus in dorsal view **22** median lobe of aedeagus in lateral view **23** male tergite VIII **24** male sternite VIII **25** female tergite VIII **26** female sternite VIII. Scale bar of habitus = 1 mm; remaining scale bars = 0.2 mm.

This species may be distinguished from other Canadian *Philhygra* by its small subparallel body (length 2.8-3.2 mm), colour dark brown with reddish or yellowish elytra and darker scutellar section, subquadrate pronotum, elytra slightly longer than pronotum, antennal articles V-X subquadrate to slightly elongate (Fig. 21), and distinctive genital structures and terminalia (Figs 22–26).

##### Distribution.

**Table:** 

Origin	Nearctic
Distribution	Canada: **AB**. USA: AZ
New records	New country and provincial record: **Canada, Alberta**: Athabasca, 19 km N Calling Lake, 55.3046°N, 113.4848W, Hammond window-trap, H-95-2-2, 1997.07.24 (NoFC) 1 male; Lacombe, La17-2002 pitfall, 52.28°N, 113.44°W, 11–18.07.2003, plot#108 back (LFC) 1 male, same data except 27.06–4.07.2003, plot#306 front (LFC) 1 male; La52-2003 pitfall, 3-10.07.2003, plot#106 (LFC) 1 female; La17-2005, 7-14.07.2005, J. Broatch (LFC) 1 male, 1 sex undetermined.
Reference	Fenyes 1909

##### Natural history.

In Alberta, adults were caught in window traps attached to aspen snags in a boreal aspen stand harvested two years previously, and in pitfall traps deployed in canola fields. Adults were collected in July.

##### Comments.

It is the first record of this species in Canada, and its broader distribution in Canada is unknown. It is probably continuously distributed in the Rocky Mountains, from Arizona in the south to Canada in the north.

### HOMALOTINI Heer

#### Agaricomorpha vincenti

Taxon classificationAnimaliaColeopteraStaphylinidae

Klimaszewski & Webster

Figs 27–33

Agaricomorpha vincenti Klimaszewski & Webster (2016).

##### Diagnosis.

**Figures 27–33.**
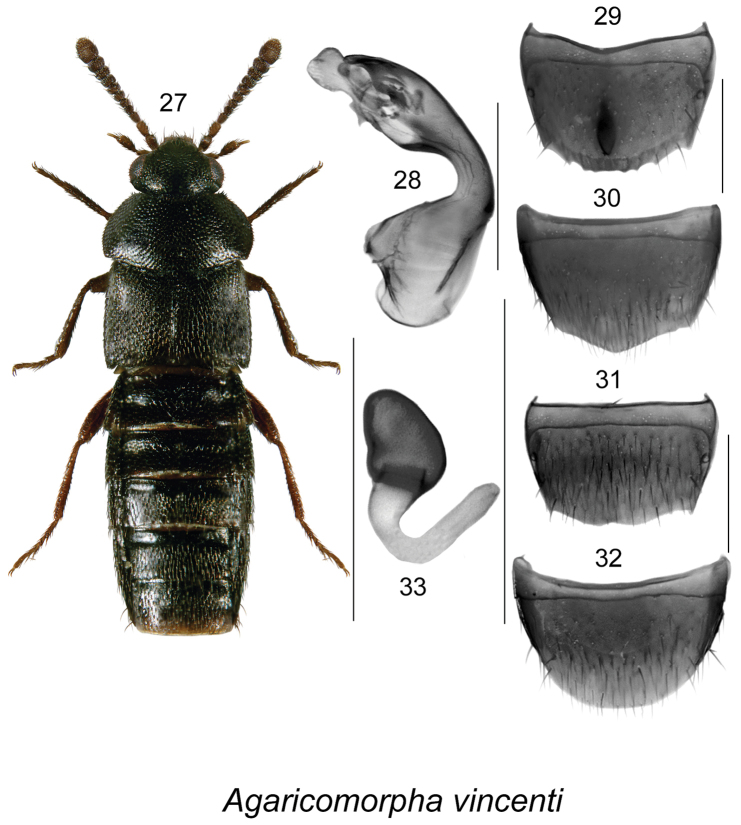
*Agaricomorpha vincenti* Klimaszewski & Webster: **27** habitus in dorsal view **28** median lobe of aedeagus in lateral view **29** male tergite VIII **30** male sternite VIII **31** female tergite VIII **32** female sternite VIII **33** spermatheca. Scale bar of habitus = 1 mm; remaining scale bars = 0.2 mm.

This species is distinguishable by its small body that is compact and narrowly oval in outline (Fig. 27); length 1.7–1.9 mm; uniformly black; forebody with strong microsculpture, that on elytra and abdomen forming scale-like structures, punctation coarse, sparse and flatly impressed, pubescence sparse and approximately evenly distributed on forebody (Fig. 27).

*Agaricomorpha vincenti* may be readily distinguished from *Agaricomorpha websteri* Klimaszewski & Brunke by the differently shaped pronotum, which is much broader than the elytra (Fig. 27), by its uniformly black body, and by the shape of median lobe of aedeagus (Fig. 28), male tergite VIII (Fig. 29), and spermatheca (Fig. 33).

##### Distribution.

**Table:** 

Origin	Nearctic
Distribution	Canada: NB, **AB**
New records	New provincial record: **Canada, Alberta**: Athabasca, 19 km N Calling Lake, 55.3046°N, 113.4848°W, Hammond window-trap, H-95-2-1, 1996.08.29, H-95-2-4, 1996.2.4, H-95-2-3, 1997.05.28 (NoFC) 1 male, 2 females; Smith, 10 km N Lawrence Lake, 55.0432°N, 113.6650°W, Hammond window-trap, H-95-3-1, 1997.08.11, H-95-3-6, 1996.09.24 (NoFC) 2 females.
Reference	Webster et al. (2016)

##### Natural history.

In New Brunswick, specimens of *Agaricomorpha vincenti* were captured in Lindgren funnel traps in a rich Appalachian hardwood forest, a *Populus tremuloides* stand with a few conifers, an old-growth northern hardwood forest, and a hardwood forest on an island in a river. In Alberta, adults were captured in window traps attached to aspen snags in a boreal aspen stand harvested two years previously. Adults were collected during May, June, and July in New Brunswick, and in May, August and September in Alberta.

##### Comments.

This species is probably continuously distributed from New Brunswick to Alberta and likely extends further to Alaska.

#### Anomognathus athabascensis

Taxon classificationAnimaliaColeopteraStaphylinidae

Klimaszewski, Hammond & Langor sp. n.

http://zoobank.org/F7A228CE-1A0B-463F-A85E-79D846E8B3F9

Figs 34–40

##### Holotype

(male). **Canada, Alberta**, Athabasca, 19 km N Calling Lake, 55.3046°N, 113.4848°W, Hammond window-trap, H-95-2-6 (CL), 1997.06.23 (NoFC). **Paratypes**. **Canada, Alberta**, Athabasca, 19 km N Calling Lake, 55.3046°N, 113.4848°W, Hammond window-trap, H-95-2-3 (CL), 1997.06.23 (LFC, NoFC) 1 female; **Canada, Alberta**, Athabasca, 19 km N Calling Lake, 55.3046°N, 113.4848°W, Hammond window-trap, H-95-2-3 (CL), 1997.07.09 (NoFC) 1 female.

##### Etymology.

*Athabascensis* is a Latin adjective derived from the name of the Athabasca region in Alberta, where the type series was discovered.

##### Diagnosis.

**Figures 34–40.**
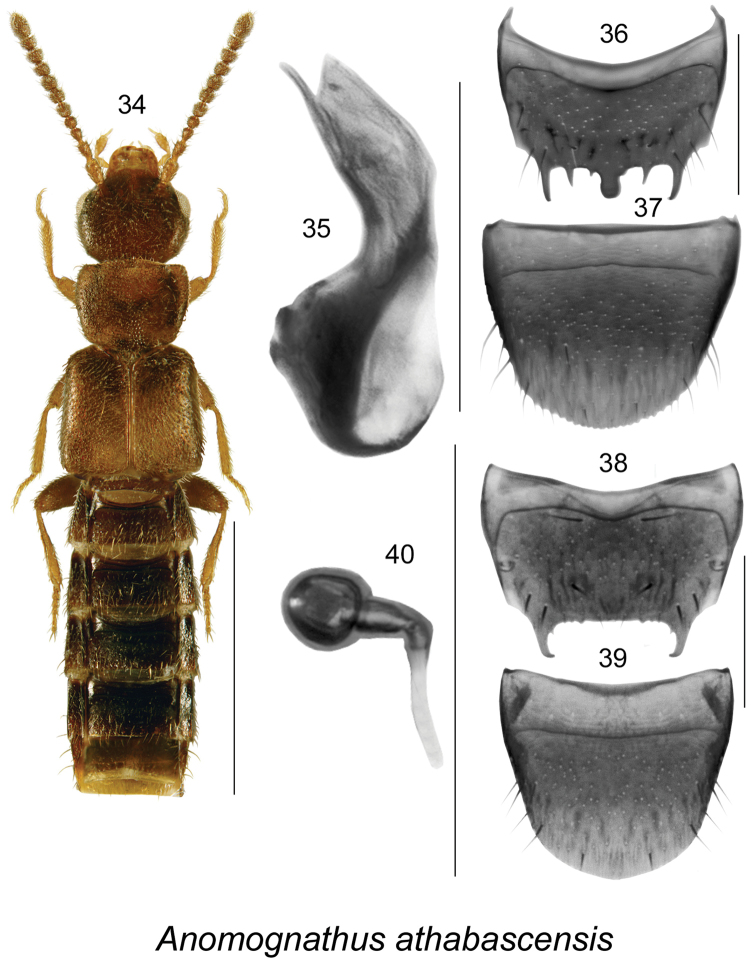
*Anomognathus athabascensis* Klimaszewski, Hammond & Langor: **34** habitus in dorsal view **35** median lobe of aedeagus in lateral view **36** male tergite VIII **37** male sternite VIII **38** female tergite VIII **39** female sternite VIII **40** spermatheca. Scale bar of habitus = 1 mm; remaining scale bars = 0.2 mm.

Body length 2.5–2.7 mm; narrow and flat (Fig. 34); more or less uniformly dark brown or reddish-brown with darker head and abdomen, with legs reddish-brown, moderately densely punctate and pubescent, pubescence short and adhering to the body, integument with dense meshed microsculpture, denser on forebody, sculpticells hexagonal, and punctation asperate on forebody; head large, rounded posteriorly and with postocular area strongly converging basally (Fig. 34), slightly wider and longer than pronotum, with small eyes shorter than postocular area; antennae with articles I-III elongate and IV-X subquadrate to slightly transverse (Fig. 34); pronotum about trapezoidal in shape, narrowest at base, widening apically to about apical third and then narrowed apically, slightly transverse, much narrower at base than elytra (Fig. 34); elytra flattened, longer than pronotum, with strong angular shoulders (Fig. 34); abdomen narrow and subparallel, paratergites well developed (Fig. 34). MALE. Median lobe of aedeagus with tubus strongly produced ventrally in lateral view (Fig. 35); internal sac without distinct sclerites (Fig. 35); tergite VIII truncate apically with three pairs of dorsal teeth and narrow median lobe (Fig. 36); sternite VIII wide, broadly rounded apically (Fig. 37). FEMALE. Tergite VIII truncate apically, with two large and hooked apically lateral teeth and some crenulation on apical margin (Fig. 38); sternite VIII rounded apically and with broad space between base of the disc and antecostal suture (Fig. 39); spermatheca with small spherical capsule and narrow and short stem (Fig. 40).

**Figures 41–44.**
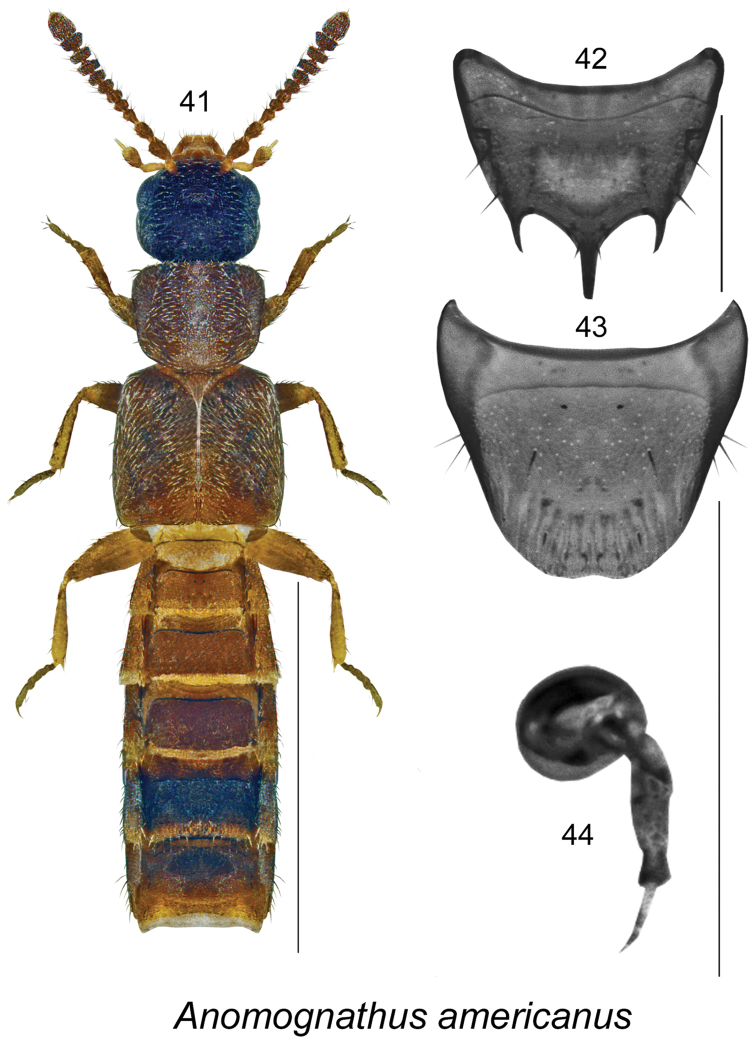
*Anomognathus americanus* (Casey): **41** habitus in dorsal view **42** female tergite VIII **43** female sternite VIII **44** spermatheca. Scale bar of habitus = 1 mm; remaining scale bars = 0.2 mm.

This species is readily distinguishable from *Anomognathus americanus* Casey, the only other representative of this genus in North America (Figs 41–44), by the different body proportions (Fig. 34), head large, longer and wider than pronotum (Fig. 34), and differently shaped tergite VIII of female (male of *Anomognathus americanus* is unknown), with two large and hooked apically lateral teeth (Fig. 38), while in *Anomognathus americanus* tergite VIII has two lateral teeth and one long median spine (Fig. 42).

##### Distribution.

Known only from Alberta, Canada.

##### Natural history.

This species was captured in June and July in Alberta. This is a subcortical species whose life history remains unknown. It is most likely associated with galleries of wood boring insects.

#### Gyrophaena sculptipennis

Taxon classificationAnimaliaColeopteraStaphylinidae

Casey

Figs 45–51

Gyrophaena sculptipennis Casey 1906: 298; Seevers 1951: 689.

##### Diagnosis.

**Figures 45–51.**
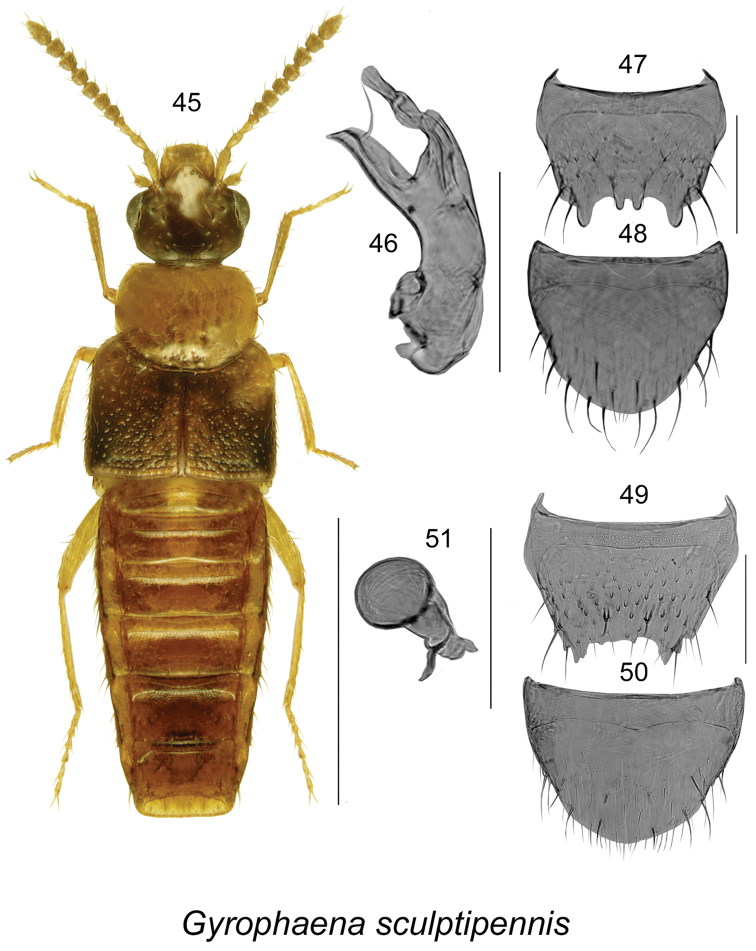
*Gyrophaena sculptipennis* Casey: **45** habitus in dorsal view **46** median lobe of aedeagus in lateral view **47** male tergite VIII **48** male sternite VIII **49** female tergite VIII **50** female sternite VIII **51** spermatheca. Scale bar of habitus = 1 mm; remaining scale bars = 0.2 mm.

This species is easily distinguishable from other *Gyrophaena* by body shape and colouration (Fig. 45), and the shape of the male and female genital structures (Figs 46–51). For a detailed description, see Seevers (1951).

##### Distribution.

**Table:** 

Origin	Nearctic
Distribution	Canada: NB, NS, QC, ON, **AB**. USA: MA, NH, NY, WI
New records	New provincial record: **Canada, Alberta**: Ft. McMurray, 35 km N Mariana Lake, 56.2821°N, 111.8337°W, Hammond window-trap, F-82-3-5 (FM), 1996.08.29 (NoFC) 1 male.
References	Casey 1906, Seevers 1951, Bousquet et al. 2013

##### Natural history.

Very little is known about the life history of this species. The Alberta specimen was captured in a window trap attached to aspen snag in a forest that burned 15 years previously. Adults were collected in Alberta in August and elsewhere in June and August (Seevers 1951).

##### Comments.

This species is probably continuously distributed from Nova Scotia and New Brunswick to the eastern Rocky Mountains.

### PLACUSINI Mulsant & Rey

#### Placusa vaga

Taxon classificationAnimaliaColeopteraStaphylinidae

Casey

Figs 52–59

Placusa vaga Casey 1911: 189, Klimaszewski et al. 2001: 27; Bousquet et al. 2013: 123.

##### Diagnosis.

This species is easily distinguishable from other Nearctic *Placusa* by its uniformly black to rarely dark brown body, long elytra (Fig. 52), and the shape of the genital structures (Figs 53–59). For a detailed description, see Klimaszewski et al. (2001).

**Figures 52–59.**
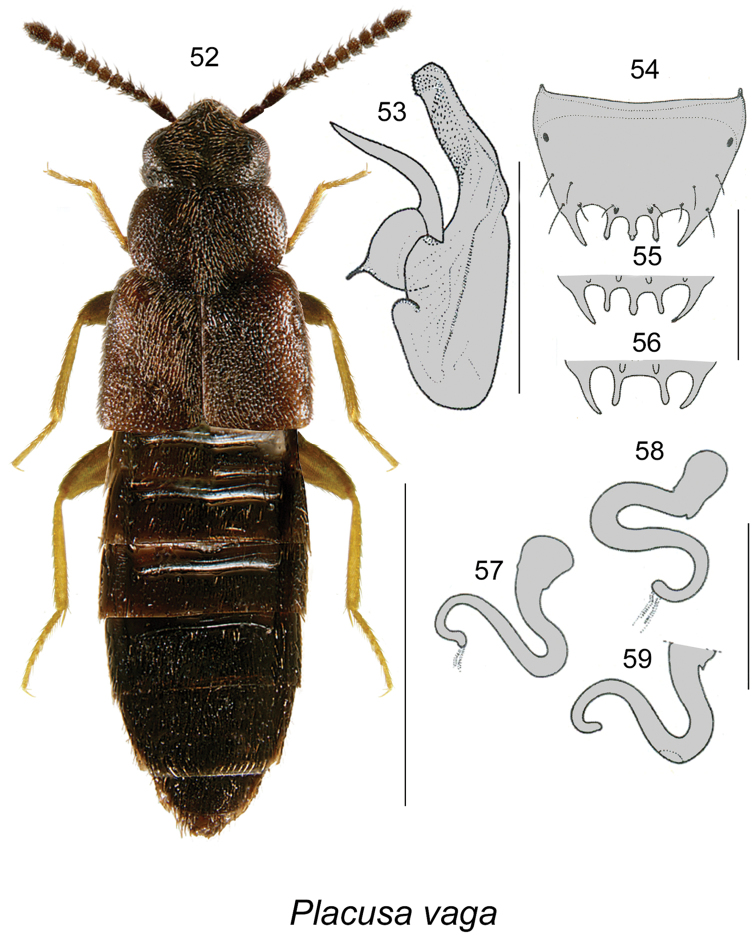
*Placusa vaga* Casey: **52** habitus in dorsal view **53** median lobe of aedeagus in lateral view **54–56** male tergite VIII **57–59** spermatheca. Scale bar of habitus = 1 mm; remaining scale bars = 0.2 mm.

##### Distribution.

**Table:** 

Origin	Nearctic
Distribution	Canada: NS, NB, QC, ON, **AB**, YT, NT, BC. USA: CA
New records	New provincial record: **Canada, Alberta**: Ft. McMurray, 15 km N Mariana Lake, 56.1848°N, 111.9513°W, Hammond window-trap F-68-1-6 (SL), H-95-3-1 (LL) D.W. Langor (NoFC) 1 male, 2 females
References	Casey 1911, Klimaszewski et al. 2011, Bousquet et al. 2013

##### Natural history.

Very little is known about the life history of this species. Adults in Quebec were captured in coniferous forests and mainly trapped in Lingren funnel traps (Klimaszewski et al. 2001). The Alberta specimens were captured in a window-traps attached to aspen snag in boreal aspen stands burned two years previously. Adults were collected in Alberta in August and elsewhere in June and August (Seevers 1951).

##### Comments.

This species is likely continuously distributed from Nova Scotia to British Columbia in northern boreal forest.

### OXYPODINI C.G. Thomson

#### Hylota cryptica

Taxon classificationAnimaliaColeopteraStaphylinidae

Klimaszewski & Webster

Figs 60–66

Hylota cryptica Klimaszewski & Webster, in Webster et al. (2016)

##### Diagnosis.

**Figures 60–66.**
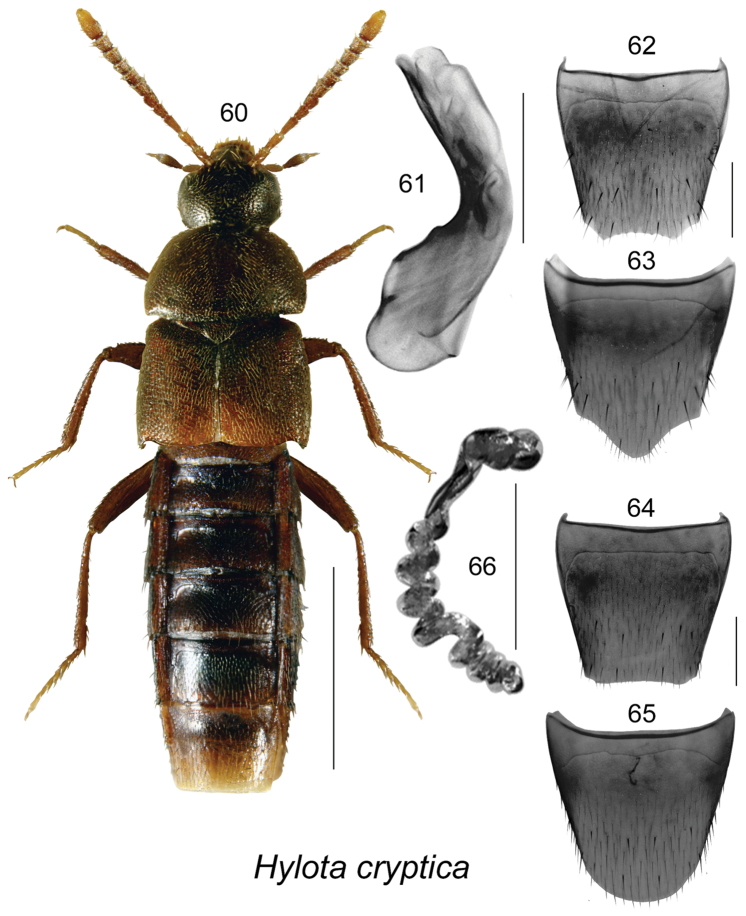
*Hylota cryptica* Klimaszewski & Webster: **60** habitus in dorsal view **61** median lobe of aedeagus in lateral view **62** male tergite VIII **63** male sternite VIII **64** female tergite VIII **65** female sternite VIII **66** spermatheca. Scale bar of habitus = 1 mm; remaining scale bars = 0.2 mm.

This species is distinguishable by length 3.2–3.4 mm, body narrowly oval, dark brown except for paler antennae, tarsi, and posterior part of elytra near suture (Fig. 60); forebody densely punctate and pubescent; head about one-third of maximum pronotal width; antennal articles IV-X from slightly elongate to subquadrate (Fig. 60); pronotum broadest at basal third and strongly narrowed apically, at base as wide as elytra (Fig. 60). *Hylota cryptica* may be separated from *Hylota ochracea* by its larger, broader and darker body, pronotum at least as wide as elytra at base (slightly narrower in *Hylota ochracea*), elongate antennal articles V-X (transverse in *Hylota ochracea*), less bent tubus of median lobe laterally (Fig. 61), apical margin of male tergite VIII with minute crenulation (Fig. 62) (with teeth in *Hylota ochracea*), and spermatheca with fewer coils (Fig. 66) (8–9 in *Hylota cryptica* and about 15–17 in *Hylota ochracea*).

##### Distribution.

**Table:** 

Origin	Nearctic
Distribution	Canada: NB, **AB**
New records	New provincial record: **Canada, Alberta**: Ft. McMurray, 15 km N Mariana Lake, 56.1848°N, 111.9513W, Hammond window-trap, F-82-3-4, 1997.06.23, F-82-3-2, 1997.06.10 (NoFC) 2 females; Slave Lake, 11 km N town Slave Lake, 55.4045°N, 114.6431°W, Hammond window-trap, H-82-3-3, 1997.06.18 (NoFC) 1 female.
References	Klimaszewski et al. 2006, Webster et al. (2016)

##### Natural history.

All New Brunswick specimens of *Hylota cryptica* were captured in Lindgren funnel traps or flight intercept traps in various forest types (Webster et al. 2016). These included a red oak forest, an old mixed forest with red oak, mixed forests, a hardwood forest on an island in a river, an old-growth northern hardwood forest, an old-growth white spruce and balsam fir forest, an old jack pine forest, an old red pine forest, and an old white pine stand (Webster et al. 2016). The Alberta specimens were captured in June in window traps attached to aspen snag in boreal aspen stands harvested and burned 15 years previously.

##### Comments.

This species is most likely continuously distributed from New Brunswick to Alberta.

## Supplementary Material

XML Treatment for Atheta (s. str.) borealis

XML Treatment for Atheta (Dimetrota) hampshirensis

XML Treatment for Atheta (Pseudota) pseudoklagesi

XML Treatment for Philhygra subpolaris

XML Treatment for Agaricomorpha vincenti

XML Treatment for Anomognathus athabascensis

XML Treatment for Gyrophaena sculptipennis

XML Treatment for Placusa vaga

XML Treatment for Hylota cryptica
